# Management of infected diabetic wound: a scoping review of guidelines

**DOI:** 10.12688/f1000research.18978.1

**Published:** 2019-05-24

**Authors:** Huidi Tchero, Pauline Kangambega, Sergiu Fluieraru, Farid Bekara, Luc Teot

**Affiliations:** 1 Trauma & Orthopaedic Surgery, CH Saint Martin, Saint Martin, Guadeloupe; 2 Endocrinology & Metabolism, CHRU de Pointe-A-Pitre, Pointe-A-Pitre, Guadeloupe; 3 Reconstructive and Plastic Surgery, CHRU Montpellier, Montpellier, France

**Keywords:** Diabetes mellitus, Infected wound, Diabetes complications, Diabetic ulcers, Diabetes management guidelines

## Abstract

**Background:** Various international guidelines and recommendations are available for management of diabetic foot infections. We present a review of the guidelines and recommendations for management of these infections.

**Methods:** A systematic literature search was conducted through MEDLINE, CENTRAL, EMBASE, LILACS, DARE, and national health bodies. Based on the review of fifteen documents, we present details on the importance of suspecting and diagnosing skin, superficial infections, and bone infections in diabetics.

**Results:** The guidelines recommend classifying the infections based on severity to guide the treatment. While antibiotics have shown the best results, other treatments like hyperbaric oxygen therapy and negative wound pressure have been debated. It is suggested that a team of specialists should be in-charge of managing the infected wounds. Infectious Diseases Society of America (IDSA) 2012 guidelines are widely followed world-over. All guidelines and reviews have consistent suggestions on the assessment of the severity of infection, diagnosis, start, selection, and duration of antibiotic therapy.

**Conclusions: **It is reasonable to conclude that the IDSA 2012 guidelines are commonly followed across the world. There is a consensus among the Australian guidelines, Canadian guidelines, IDSA 2012, National Institute for Health and Care Excellence (NICE) 2015, and International Working Group on the Diabetic Foot (IWGDF) 2016 guidelines on the management of infected wounds for patients with diabetes mellitus.

## Introduction

Diabetes mellitus (DM) is one of the major public health issues of this century ^1^. With an increasing life expectancy, the incidence of complications in diabetics is on the rise ^2, 3^. Diabetic foot ulcers and infections affect approximately 15% of diabetic patients ^4, 5^. An infected foot is a serious complication of diabetes ^6^ and it is a factor in half of all cases of lower extremity amputations ^7^.

Various guidelines and recommendations by international health bodies and scientific associations, in addition to several systematic reviews and Cochrane reviews, are currently available to guide the selection of the correct treatment modality for infected diabetic foot ulcers/wounds ^1, 8– 10^.

There is a general lack of understanding on the infected diabetic wound management guidelines. Further, a comparison of these guidelines is necessary to understand the strengths and weaknesses of these guidelines. Hence, we believe that there was a need to conduct a scoping review to analyze the guidelines that are in practice. The purpose of this scoping review was to study the management practices currently being followed for infected diabetic wounds and present a comparative evaluation of the published guidelines and reviews.

## Methods

### Criteria for considering studies for this review

*Types of studies*

- Guidelines, recommendations or reviews from associations related to diabetes (American Diabetes Association, WHO or any regulatory body) published in English since 2000 and before December 2017 were eligible for inclusion. All the associations that have published guidelines were eligible for inclusion.

*Types of participants*

- Adults and children with DM

*Types of outcome measures*

- Management of infected wounds among patients with DM
- Antibiotic therapy

### Search methods for identification of studies

The following databases were searched on 6 ^th^ August 2016 using the search terms detailed in Table 1. The databases searched were Cochrane Central Register of Controlled Trials (CENTRAL), MEDLINE (January 2000 to July 2016), EMBASE, LILACS, The Database of Abstracts of Reviews of Effects (DARE), American Diabetes Association (ADA), European Diabetes Association, WHO, National Institute for Clinical Excellence (NICE) databases and Google Scholar. Clinical trial registries were not searched as the search was for published articles only.

**Table 1.** Details of the literature search. We searched Cochrane Central Register of Controlled Trials (CENTRAL), MEDLINE, LILACS, The Database of Abstracts of Reviews of Effects (DARE), American Diabetes Association (ADA) and European Diabetes Association on 6th August 2016.

Search set	CENTRAL	MEDLINE	LILACS	DARE	EMBASE
1	#1 diabetes	“Diabetes” as a MeSH major topic	Diabetes - title	Diabetes foot ulcers	#1 diabetes/exp
2	#2 wound	Infections MeSH term	Wound – title/ abstract		#2 diabetes
3	#3 infection	Wound – title/abstract	Infection- title/abstract		#3 wound/exp
4	#4 ulcer				#4 wound
5	#4 OR #2				#5infection/exp
6	#1 and #3 and #5				#6 infection
7					#1 OR #2
8					#3 OR #4
9					#5 OR #6
10					#7 AND #8 AND #9

### Data collection and analysis

All the abstracts and titles of the studies identified by the search were scanned by two authors independently (HT and FB) for relevance according to the inclusion criteria. In the first round, publications were screened using the information in the title and abstract. In the second round, full-texts of the articles identified in the first round were studied to confirm the eligibility. Any differences in opinion about the selection of articles were resolved by a third party (LT).

### Data extraction

Two authors (HT and FB) independently retrieved relevant patients’ and intervention details using standardized data extraction forms using excel sheets. Data were collected under the following headings: Title, Year of publication, Publisher and the following variables of interest: Pathogenesis of diabetic infected wound, Diagnostic guidelines, Diagnosis of osteomyelitis, Antimicrobial treatment, Debridement, Role of other treatment modalities, Requirement for hospitalization, Role of surgery, Wound care, General considerations for management. Disagreements between reviewers, if any, were resolved through discussion to obtain a consensus.

### Reporting guidelines

This report was prepared as per the PRISMA-ScR (Preferred Reporting Items for Systematic reviews and Meta-Analyses extension for Scoping Reviews) checklist.

## Results

### Search results

**Figure 1.**
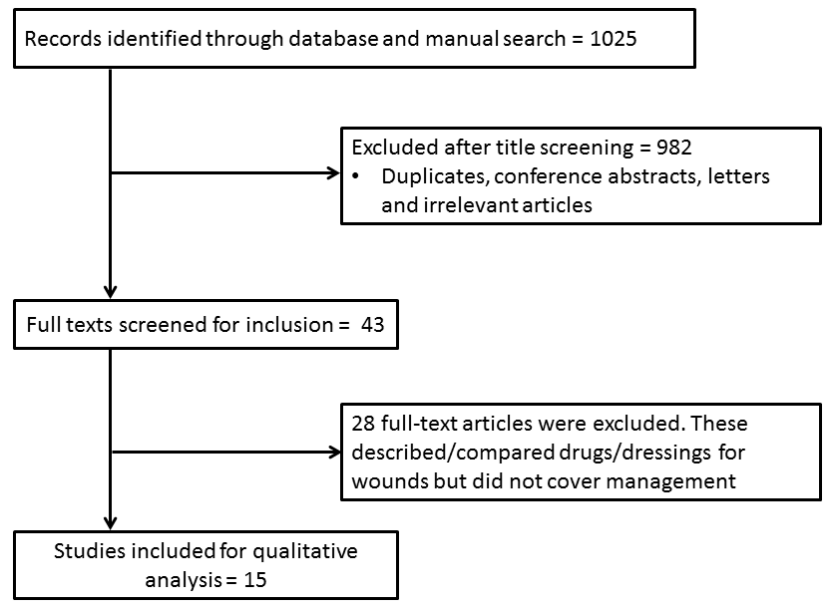
PRISMA flow diagram. PRISMA flow diagram summarizing identification, screening and inclusion of studies.

The initial search yielded 1025 abstracts, which were screened for potential inclusion in the review. After screening the abstracts, 982 were excluded due to duplication or irrelevance. A total of 15 reports/reviews were included for the scoping review (see Figure 1 for PRISMA flow diagram) ^11^. The initial plan was to compare the management practices for infected diabetic wounds in different countries around the world. However, most of the research and literature were on infected diabetic foot ulcers/wounds. Since diabetic foot ulcers are the most common presentation of diabetic infected wounds, the search results were confined to infected diabetic ulcers. During the search for management practices being followed in different parts of the world, all of the randomized controlled trials (RCTs) that compared different treatment approaches used in the management of infected diabetic wounds were excluded. Moreover, all case reports, case series, observational studies and cohort studies were excluded. Cochrane Reviews were also excluded because each review has taken into consideration one treatment modality. However, systematic reviews and descriptive reviews on different treatment options were considered.

In the next stage, guidelines and recommendations issued by various countries around the world and management review articles were evaluated. Our review included the 2004 ^10^ and 2012 guidelines of the Infectious Diseases Society of America (IDSA) ^12^, "Management of Diabetes" guidelines by the Scottish Intercollegiate ^13^, the International Working Group on the Diabetic Foot (IWGDF) review of systematic reviews ^14^, a clinical update by Australian Diabetes Foot Network ^8^, guidelines for the management of infected ulcers among patients with DM in Portugal ^15^, the Canadian Diabetes Association Clinical Practice Guidelines ^16^, and independent guidelines published by Wounds International in 2013 ^7, 9, 17– 22^. A summary of these guidelines is presented in Table 2.

**Table 2.** Summary of the guidelines included in the study.

	Guideline – DOI/URL	Key Results
1 and 2	2004 Diagnosis and Treatment of Diabetic Foot Infections and 2012 Infectious Diseases Society of America Clinical Practice Guideline for the Diagnosis and Treatment of Diabetic Foot Infections – Infectious Diseases Society of America (IDSA), https://doi.org/10.1086/424846 and https://doi.org/10.1093/cid/cis346	• Most of the diabetic foot infections (DFIs) can be cured with proper management. • DFI should be defined by presence of inflammation or purulence. • DFI should be classified based on severity. • Clinical decisions should be made based on definition and severity of DFI. • Staphylococci are the most common cause of DFI. • Definitive antibiotic therapy should be based on cultures of infected diabetic wound. • Imaging is recommended to detect osteomyelitis. • Surgery and wound care may be needed in successful cure of DFI. • Patients with DFIs should be evaluated for an ischemic foot. • Multidisciplinary approach should be employed.
3	2010 (updated 2017) Management of diabetes: A national clinical guideline - Scottish Intercollegiate Guidelines Network, https://www.sign.ac.uk/assets/sign116.pdf	• Patients with active diabetic foot disease should be referred to a multidisciplinary diabetic foot care service. • Treatment of a patient with an infected diabetic foot ulcer and/or osteomyelitis should be commenced immediately with an antibiotic in accordance with local or national protocols. Subsequent antibiotic regimens may be modified with reference to bacteriology and clinical response.
4	2012 A systematic review of the effectiveness of interventions in the management of infection in the diabetic foot – International Working group on the Diabetic Foot, https://doi.org/10.1002/dmrr.2247	• There was no better response with any particular antibiotic regimen. • No particular route of delivery or duration of treatment was found to be superior. • Hyperbaric oxygen therapy was not useful.
5	2012 Australian Diabetes Foot Network: management of diabetes-related foot ulceration — a clinical update, https://doi.org/10.5694/mja11.10347	• Appropriate assessment and management of diabetes-related foot ulcers (DRFUs) is essential to reduce amputation risk. • Management requires debridement, wound dressing, pressure off-loading, good glycemic control and potentially antibiotic therapy and vascular intervention. • As a minimum, all DRFUs should be managed by a doctor and a podiatrist and/or wound care nurse. • Health professionals unable to provide appropriate care for people with DRFUs should promptly refer individuals to professionals with the requisite knowledge and skills. • Indicators for immediate referral to an emergency department or multidisciplinary foot care team (MFCT) include gangrene, limb-threatening ischemia, deep ulcers (bone, joint or tendon in the wound base), ascending cellulitis, systemic symptoms of infection and abscesses. • Referral to an MFCT should occur if there is lack of wound progress after 4 weeks of appropriate treatment.
6	2013 Guidelines for treatment of patients with diabetes and infected ulcers – Mansilha and Brandão, https://www.ncbi.nlm.nih.gov/pubmed/23443604	• Diabetic foot infections can be classified in mild, moderate and severe according to local and systemic signs. • Their identification should lead to a prompt and systematic evaluation and treatment, ideally performed by a multidisciplinary team. • Decisions concerning empirical initial antibiotic agent(s), desirable route of administration, duration and need of hospitalization should be based on the more likely involved pathogen(s), the severity of the infection, the ulcer chronicity and the presence of significant ischemia. • Wound cultures, ideally from ulcer tissue, are strongly advisable and can help guiding and narrowing the antibiotic spectrum. • Appropriate wound care and off-loading should not be neglected. • When revascularization is required, the correct timing can be crucial for limb salvage. • Since the recurrence of ulcer and infection is high, the implementation of appropriate preventive measures can be critical. • Ultimately, the definitive goal in the treatment of diabetic foot infections is to prevent the amputation catastrophe.
7	2013 (updated 2018) Foot Care - Diabetes Canada Clinical Practice Guidelines Expert Committee, https://doi.org/10.1016/j.jcjd.2017.10.020	• People with diabetes who develop a foot ulcer or show signs of infection even in the absence of pain should be treated promptly by an interprofessional health-care team when available with expertise in the treatment of foot ulcers to prevent recurrent foot ulcers and amputation. • There is insufficient evidence to recommend any specific dressing type for typical diabetic foot ulcers. • Debridement of nonviable tissue and general principles of wound care include the provision of a physiologically moist wound environment and off-loading the ulcer. • There is insufficient evidence to recommend the routine use of adjunctive wound-healing therapies (e.g. topical growth factors, granulocyte colony-stimulating factors or dermal substitutes) for typical diabetic foot ulcers. Provided that all other modifiable factors (e.g. pressure off-loading, infection, foot deformity) have been addressed, adjunctive wound-healing therapies may be considered for nonhealing, nonischemic wounds.
8	2013 Best practice guidelines: Wound management in diabetic foot ulcers - Wounds International, https://www.woundsinternational.com/download/ resource/5958	• Management of an infected diabetic ulcer should be aimed at preventing life- or limb- threatening complications • For superficial (mild) infections — treat with systemic antibiotics and consider topical antimicrobials in selected cases • For deep (moderate or severe) infections — treat with appropriately selected empiric systemic antibiotics, modified by the results of culture and sensitivity reports • Offload pressure correctly and optimize glycemic control for diabetes management • Consider therapy directed at biofilm in wounds that are slow to heal

### Quality of the reviews included

As our scoping review included guidelines, descriptive reviews and systematic reviews, we didn’t use the Assessment of Multiple Systematic Reviews (AMSTAR) tool, which is generally used for quality assessment of the included systematic reviews ^23^. The systematic reviews by Braun *et al.* (2012) and Peters *et al.* (2016) had included RCTs after searching PubMed and EMBASE ^14, 17, 21^. The review by Wukich *et al.* is a descriptive review without any systematic searches of databases and, of note, the authors had received grants from pharmaceutical companies ^22^. Studies by Joseph and Lipsky, Mansilha and Brandao, and Gemechu *et al.* were also narrative reviews without any systematic search of the databases ^7, 15, 19^.

### Outcome measurements

***Pathogenesis.*** The guidelines highlighted that the most common causative organisms of diabetic infections are aerobic gram-positive cocci, especially *Staphylococcus aureus*, including methicillin-resistant strains (MRSA), and *Streptococcus spp.* (most often Group B). Chronic infections or those occurring after antibiotic treatment are generally polymicrobial, with aerobic gram-negative bacilli and anaerobes. Obligate anaerobes are isolated more commonly from cases of foot ischemia or necrosis. In southern European countries, gram-negative bacilli are more prevalent ^9, 15, 16, 19, 22^.

***Diagnosis of the diabetic infected wound.*** All the guidelines, including IDSA 2004, IDSA 2012, IWGDF 2012, and IWGDF 2016 and those by Wounds International Society, recommend that diagnosis of infections should be made on the basis of clinical signs and symptoms ^9, 10, 12, 14, 21^. According to the IDSA 2004 guidelines, the diagnosis of infected wounds should be made clinically on the basis of local (and occasionally systemic) signs and symptoms of inflammation ^12, 19^. Joseph *et al.* mentioned that patients should have clinical signs and symptoms of inflammation such as erythema, edema, warmth, tenderness, indurations and/or pain ^19^. The IDSA 2012 guidelines recommend that diagnosis of infections should be based on the presence of at least two classic symptoms or signs of inflammation (erythema, warmth, tenderness, pain or indurations) or purulent secretions. This recommendation was also emphasized by other reviewers ^7, 10, 22^.

***Suspicion/diagnosis of osteomyelitis.*** The IDSA 2012 guidelines and NICE recommendations (2015) strongly recommend that clinicians should suspect osteomyelitis as a complication of diabetic foot infections (DFIs) ^10, 20^. In earlier guidelines, microbiological and laboratory investigations were recommended for the diagnosis of osteomyelitis ^12^. The IDSA 2012, Wounds International guidelines and Australian guidelines suggest that culture and histology findings help with diagnosing osteomyelitis. Due to the unavailability of these tests at many places, clinicians should make a diagnosis in conjunction with clinical, radiographic and laboratory findings ^8– 10^. An increase in skin pigmentation may be considered a sign of inflammation and/or infection among patients with pigmented skin ^8^. All guidelines recommend magnetic resonance imaging (MRI) as the best imaging technique to define both soft tissue and bone infections ^8– 10, 20^. Plain radiography is considered to be less sensitive for DFIs; however, it may be helpful in assessing bone destruction and the presence of gases or foreign bodies ^9, 10, 22^. As the radiological signs of osteomyelitis are delayed, a normal report resulting from a plain X-ray should be cautiously interpreted ^9^. The IDSA 2012 guidelines strongly recommend that all patients with a new DFI should have a plain radiograph of the affected foot to check whether bones are affected, and whether gases or foreign bodies are present ^10^. Probe-to-bone testing, an inexpensive diagnostic tool, may be helpful in confirming the diagnosis ^7^. Bone samples for culture and histology should be taken after debridement or by bone biopsy ^10^. In addition, white blood cell scanning combined with a radionuclide bone scan can be performed to assist diagnosis ^9^. After the diagnosis of an infected wound and presence or absence of osteomyelitis, it is equally important to classify the severity of the infection, as the treatment choice depends on the severity.

***Antimicrobial treatment.*** Appropriate culture samples should be taken, preferably from soft tissue or purulent secretions, for appropriate selection of antibiotic to be used ^8, 9, 20^. Tissue specimens or deep swabs should be cultured for both aerobic and anaerobic organisms ^9^. Superficial sampling can miss the true causative organism, thus deep sampling after cleansing or debridement can be helpful ^9^. All guidelines recommend that clinically uninfected ulcers should not be treated with antibiotic therapy. It is strongly recommended that no topical or systemic antibiotic therapy should be given to prevent osteomyelitis, improve wound healing or prevent secondary infection ^8– 10, 18, 20^. Moreover, NICE guideline (2015) also suggested that antibiotic treatment should be started as soon as possible. Culture samples should be taken before the start of the treatment ^20^. NICE guideline (2015) provides wide criteria to choose the appropriate antibiotic and the regimen, such as the severity of the infection, care setting, person’s preference, clinical situation, medical history, microbiological examination, clinical response and cost ^20^. However, tigecycline should not be offered unless other antibiotics are not suitable ^20^. The IDSA 2012, NICE 2015, Wounds International and Scottish guidelines specified that the duration and route of the antibiotic administration should be based on the severity of the disease, presence or absence of bone infection and clinical response to treatment ^9, 10, 13, 20^. In the case of neuroischemic foot ulcer, antibiotics should be chosen carefully as it is more serious than an neuropathic foot ulcer ^9^.

The IDSA 2012 guidelines stated that the U.S. FDA has approved three antibiotics (ertapenem, linezolid and piperacillin-tazobactum) for the treatment of complicated skin and skin structure infections including DFIs, but not for accompanying osteomyelitis ^10^. However, there is no evidence for the superiority of any particular antibiotic agent, treatment duration or route of administration ^10, 13, 14^. Wukich *et al.* mentioned that no one agent or regimen has shown superiority over others; however, those that have demonstrated effectiveness include β lactams (penicillins, cephalosporins), glycopeptides, carbapenems, linezolid, clindamycin and fluoroquinolones ^22^. There is weak evidence to suggest that antibiotic therapy against bone culture leads to higher resolution of bone infection compared to that of empiric therapy ^10^. Also, IDSA 2012 guidelines suggest that antibiotic therapy should be continued only until the resolution of infection ^10^.

*Empiric therapy:* IDSA 2004 and 2012 strongly recommend that an empiric antibiotic regimen should be chosen on the basis of the severity of the infection and the likely etiologic agent(s) ^10, 12, 19^. The guidelines recommend a broad-spectrum antibiotic for severe cases, whereas a narrow spectrum antibiotic should be used for mild cases. The antibiotic agent can be modified following culture reports and antibiotic susceptibility data ^8– 10, 16, 19, 22^. The IDSA 2004 guidelines highlighted the importance of escalation and de-escalation regimes depending upon the culture reports ^12^. The local prevalence of MRSA strains should determine the choice of empiric therapy ^19^. Empiric antibiotic therapy against MRSA should be given to patients with a prior history of MRSA infections, or in instances with high local MRSA prevalence colonization, or in cases where the infection is severe ^9^. IDSA 2012 highly recommended that empiric therapy directed at *Pseudomonas aeruginosa* is usually not required except among those patients with risk factors for *Pseudomonas aeruginosa* infection ^10^. Recommendations from Wounds International suggest that empiric oral antibiotic therapy against *Staphylococcus aureus* and *β hemolytic Streptococcus* are given in the case of mild infections ^9^.

*Definitive therapy:* Definitive therapy depends on the culture and sensitivity results of the wound specimen, and on the patient’s clinical response to the empiric regimen ^10^.

*Mild diabetic foot infections*: The guidelines recommend oral antibiotics with a spectrum of activity against gram-positive organisms ^8– 10, 20^. The treatment should last no longer than 14 days for mild soft tissue infections ^9, 14, 15^. Wounds International suggests that empiric antibiotic treatment should be changed according to the culture reports. Topical antibiotics can be given along with oral agents. However, topical antibiotics should not be used alone for patients with clinical signs of infection ^9^.

*Moderate diabetic foot infections*: Antibiotic agents against gram positive and gram negative organisms, including anaerobic bacteria, should be administered ^10, 20^. The route of administration should depend on the clinical condition and the availability of the antibiotic agents ^10, 20^. Recommendations from Wounds International suggest that treatment lasting one to three weeks should be sufficient; however, no specific time is allocated as each decision must be based on the severity and clinical response of the patient ^9^. Other guidelines have also suggested similar periods of 2–3 weeks or 2–4 weeks ^10, 15, 20^. The empiric antibiotic agent should be changed according to the culture reports or if the signs of inflammation do not improve ^8, 9, 13, 15, 16, 19^.

*Severe diabetic foot infections*: Intravenous administration of antibiotic agents against gram-positive and gram-negative organisms, including anaerobic bacteria, should be elicited. The treatment can be switched to the oral route depending upon the culture results and the condition of the patient ^9, 10, 20^.

*Osteomyelitis*: Surgical resection or debridement may be required in these cases. Generally, antibiotic therapy must be given parenterally and the duration of antibiotic treatment can last up to six weeks. There is no evidence of superiority of any group of antibiotics or their route of administration over others ^8– 10, 16, 20^.

*Topical antibiotic therapy*: Although there is no robust evidence to support the use of topical antimicrobials, especially topical antiseptics (such as cadexomeriodine) and silver-based dressings, they are currently being used to decrease the bio-burden of the wound ^10^. However, they may increase the risk of bacterial resistance in addition to causing local adverse effects. The IDSA 2012 guidelines recommended neither the use of topical antimicrobials for most clinically uninfected wounds nor silver-based dressings for clinically infected wounds ^10^. Wounds International suggests that topical antimicrobials may be used alone (but not in patients with clinical signs of infection) or as an adjuvant therapy when there are concerns regarding reduced antibiotic tissue penetration, such as patients with poor vascular supply, and in non-healing wounds with no signs and symptoms of infection, but with increased bacterial bio-burden ^9^. In these cases, topical antimicrobials may prevent the spread of infection to deeper tissues ^9^. Regular monitoring is required to check for improvement and to inform decisions on whether to continue or withdraw treatment ^9^.

***Debridement.*** Wounds International states that mild diabetic infections may require debridement and wounds should be cleaned with saline during the application of every dressing ^9^. The formation of biofilms can be controlled by repeated debridement and cleansing ^9^. IDSA 2012 strongly recommends debridement to remove debris, eschar, or surrounding callus. Sharp debridement methods are considered to be the best; however, other methods such as mechanical, autolytic or larval debridement may be useful in some settings ^10, 18^.

***Role of other treatment modalities.*** So far, there has been insufficient data to support the use of other treatment modalities.

*Granulocyte colony stimulating factors*: There is a weak recommendation for its use as an adjunctive treatment; adding G-CSF did not affect the resolution of infection or the duration of systemic antibiotic therapy ^10, 14, 16, 17^.

*Hyperbaric oxygen therapy (HBOT)*: Guidelines and reviews are not in favor of the use of HBOT for ulcer healing, mainly due to the controversial data relating to it ^14, 16, 17^.

*Negative pressure wound therapy (NPWT)*: This should be considered in patients with active diabetic foot ulcers or postoperative wounds ^13^. Braun *et al.* stated that there is a moderate level of evidence in favor of using NPWT to heal diabetic foot ulcers ^17^. IWGDF 2016 also suggested that there is a weak evidence in favor of using NPWT in post-operative wounds, although its effectiveness and cost-effectiveness remains unknown ^21^.

*Topical antiseptic agents*: There is a weak evidence for the use of topical antiseptics such as super oxidized water or iodophor to decrease cellulitis ^14^. The IWGDF 2016 guidelines state that as a result of poor trial designs, it is difficult to draw conclusions in favor of or against the use of topical treatments with antiseptic agents ^21^. The latest IWGDF 2016 recommendations demonstrate little evidence in favor of using honey as an antibiotic agent ^21^. The IDSA 2012 guidelines demonstrate a moderate level of evidence and provide weak recommendations for other modalities such as bioengineered skin equivalents, growth factors, and negative pressure wound therapy ^10^.

***Requirement for hospitalization.*** The IDSA 2012 strongly recommended that patients with a severe infection, some patients with a moderate infection, those who are unable to comply with outpatient treatment, and those with poor response to the therapy should be hospitalized initially ^10, 22^.

***Role of surgery.*** Surgical consultation is required for deep abscesses, gas in deeper tissues, extensive bone or joint involvement, gangrene or necrotizing fasciitis ^7, 10, 19^. Evidence from former systematic reviews demonstrated that early surgery decreases the requirement for amputation significantly in two single center studies ^14, 21^.

***Wound care.*** Antibiotic therapy is necessary for virtually all infected wounds, but it is often insufficient without appropriate wound care ^19^. Dressings should be chosen according to the nature, depth and size of the ulcer ^10^. Regular monitoring, involving radical and repeated debridement, frequent inspection and bacterial control, are important measures in this regard ^9^.

*Off-loading*: Off-loading is essential for diabetic foot management. According to a consensus guideline at Journal of the American Podiatric Medical Association, there is a strong evidence that adequate off-loading increases the likelihood of diabetic foot ulcer (DFU) healing ^24^. Nonremovable casts or fixed ankle walking braces are currently perceived as optimum off-loading modalities. However, a gap still exists between what the evidence suggests and what is being performed in clinical practice.

***General considerations for management.***

*Role of teams and specialists*: All guidelines stressed the importance of having teams of specialists treating diabetic infected wounds ^8– 10, 22^. Specialists should be sought if the attending physician is not familiar with the techniques of wound care ^10^. Diabetic foot care teams can include (or should have ready access to) specialists in various fields ^10^. It is strongly recommended to consult a vascular surgeon for revascularization for patients with evidence of ischemia of the infected limb ^10^. Similarly, Wounds International suggested surgical consultation for rapidly deteriorating wounds that do not respond to antibiotic therapy ^9^. Glycemic control is also important during the management of diabetic infected wounds as the correction of hyperglycemia may lead to a favorable response ^8– 10, 22^. Lipid and blood pressure levels should be within control, and smoking cessation should be advised ^8^.

*Patient education*: Being the primary care-givers for their own feet, patients should be aware of the risk factors that could predispose to or worsen DFIs and the appropriate care and management behaviors. Former studies showed that patient education programs could be of substantial benefit in reducing the incidence of DFUs and improving self-care practices ^25^. Patients should be taught to examine their feet daily and report any abnormality to their physician, trim toenails with a safety clipper and wear offloading casts. Moreover, patients should be aware of the importance of exercise, smoking cessation for smokers and compliance to diabetes control instructions ^26, 27^.

*Amputation*: Although not a preferred treatment approach, amputation may be required in certain situations, such as life-threatening foot infections that cannot be managed by other measures, non-healing ulcers with a disease burden higher than expected after amputation or where ischemic rest pain cannot be managed by analgesia or revascularization ^9, 10, 22^.

## Discussion

This scoping review aimed to compare the management practices currently being followed in different parts of the world to treat diabetic infected wounds. As described in the results section, research is ongoing to decide appropriate management of diabetic infected wounds. The literature search identified the guidelines/recommendations and systematic reviews published on the management of diabetic infected wounds from 2000 to August 2016. The aim to consider the global practices was achieved, as recommendations from North America ^10, 16, 22^, Europe ^13, 15, 20^, Australia ^8^, and International scientific societies ^9, 18^ were included.

Our review highlights that the guidelines across the world provided similar recommendations for the management of infected diabetic wounds. The first stage of suspecting and diagnosing infections was emphasized by all the guidelines. The IDSA 2004 guidelines recommended diagnosis based on the presence of clinical symptoms and signs of local inflammation; however, the IDSA 2012 guidelines recommended diagnosis based on the presence of two clinical symptoms and signs of local inflammation ^10, 12^. There is a consensus among the guidelines on the requirement to suspect and quickly diagnose osteomyelitis ^10, 18, 21^. MRI was established as the best diagnostic method, while plain X-ray should not be considered for diagnosis ^9, 10, 20^.

All the included reviews and guidelines have concluded that most acute infections are caused by gram-positive cocci, *S. aureus* and *Streptococci*, and that gram-negative cocci or anaerobes may be involved, as infections are generally polymicrobial ^8– 10, 15^. The IDSA 2012 clinical practice guideline has suggested the use of any of three antimicrobials (ertapenem, linezolid, and piperacillin-tazobactum) for diabetic infected wounds; however, there is a consensus on the non-superiority of any one antibiotic agent over the other two ^9, 10, 13^. Similarly, there is a consensus on the importance of local protocols, the prevalence of MRSA, and the severity of the wound as important deciding factors while selecting the appropriate antibiotic therapy ^9, 10^. NICE 2015 further highlighted the importance of cost when choosing the antibiotic agent ^20^. The severity classification of IDSA 2012 has been accepted universally ^10^. There are similar recommendations for the choice of empiric therapy and the duration of the antibiotic therapy across all included reviews.

The guidelines did not suggest anti-microbial use for clinically uninfected wounds ^8– 10, 20^. Other similar recommendations include the importance of regular monitoring and the crucial role of multidisciplinary teams consisting of microbiologists, infectious disease specialists, surgeons and medical specialists ^8, 10, 15^.

The guidelines and reviews provided strong recommendations on the previously mentioned modalities; however, there is weak evidence to support some routinely followed treatment practices. With respect to hyperbaric oxygen therapy, the latest guidelines provide weak evidence to support the use of alternative modes such as hyperbaric oxygen or NPWT ^8– 10, 20, 22^. However, this modality requires further exploration and research. A retrospective study of patients with DM with hand infections demonstrated that the addition of HBOT to standard therapies is safe due to its anti-infective effects ^28^.

Regarding wound care, the analysis demonstrates that it is equally as vital as the use of antibiotic agents. However, again, there is a lack of evidence in favor of the many available wound care modalities. At the same time as IDSA and NICE guidelines were released, various systematic reviews have sought to evaluate the options of wound care. Alginate dressings, foam dressings and hydrocolloid dressings were not found to promote the healing of diabetic foot ulcers any better than other alternative dressings. The reviews concluded that the decision-makers may consider other aspects such as cost and wound management properties when selecting the dressing type ^2, 29^. Another systematic review by Cochrane showed that NPWT may be an effective treatment to heal debrided foot ulcers and post-operative amputation wounds; however, the studies included could be at risk of bias ^30^.

While conducting this scoping review, all English guidelines published by the previously mentioned associations from around the world since 2000 were included. Strengths of this scoping review are the inclusion of recommendations from different corners of the world and an extensive search of various databases. However, the inclusion of only English language published reviews limited the search. It was not possible to calculate the quality of all the articles included as some were guidelines that could not be assessed by the scales available.

## Conclusion

It is reasonable to conclude that the IDSA 2012 guidelines are commonly followed across the world. There is a consensus among the Australian guidelines, Canadian guidelines, IDSA 2012, NICE 2015 and IWGDF 2016 guidelines on the management of infected wounds for patients with DM. Due to the lack of evidence, the therapeutic status of treatment options like hydrocolloid gels, NPWT, hyperbaric oxygen and aligate dressings could not be ascertained. There is a need to generate stronger evidence regarding the commonly used methods in the treatment of diabetic wound infections.

## Data availability

### Underlying data

All data underlying the results are available as part of the article and no additional source data are required.
