# Supplementary material for: Management of infected diabetic wound: a scoping review of guidelines
Source: F1000Res. 2019 May 24;8:737. [Version 1] doi: 10.12688/f1000research.18978.1 (PMC7265589; doi:10.12688/f1000research.18978.1)
Supplement: Peer review history

# Peer review: Reviewer response for version 1

**DOI:** 10.5256/f1000research.20803.r63740

**Published:** 2020-06-01

**Authors:** Muhammad Bilal Tahir

**Affiliations:** 1 Department of Physics, Faculty of Science, University of Gujrat, Gujrat, Pakistan

The authors comprehensively discussed the diabetic patients, their complications, and recommended the future guidelines. A thorough study was conducted using MEDLINE, CENTRAL, EMBASE, LILACS, DARE, and national health bodies. Additionally, importance of suspecting and diagnosing skin, superficial infections, and bone infections in diabetics have been studied. I strongly recommend it.

Is the work clearly and accurately presented and does it cite the current literature?

Yes

If applicable, is the statistical analysis and its interpretation appropriate?

Not applicable

Are all the source data underlying the results available to ensure full reproducibility?

Yes

Is the study design appropriate and is the work technically sound?

Yes

Are the conclusions drawn adequately supported by the results?

Yes

Are sufficient details of methods and analysis provided to allow replication by others?

Yes

Reviewer Expertise:

Infection diseases; Diabetic patients; Nanotechnology in medicine

I confirm that I have read this submission and believe that I have an appropriate level of expertise to confirm that it is of an acceptable scientific standard.

# Peer review: Reviewer response for version 1

**DOI:** 10.5256/f1000research.20803.r63468

**Published:** 2020-06-01

**Authors:** Davoud Dastan

**Affiliations:** 1 School of Materials Science and Engineering, Georgia Institute of Technology, Atlanta, GA, USA

This review by Huidi Tchero *et al.* reports ‘Management of infected diabetic wound: a scoping review of guidelines’. The authors claimed that the results are encouraging to develop a plan for diabetic wound and eco-friendly approach. This article signifies the importance of suspecting and diagnosing skin, superficial infections, and bone infections in diabetics. The suggested guidelines seem consistent with the suggestions on the assessment of the severity of infection, diagnosis, start, selection, and duration of antibiotic therapy. The authors further claimed that the IDSA 2012 guidelines are commonly followed across the world and there is a consensus among the Australian guidelines, Canadian guidelines, IDSA 2012, NICE 2015 and IWGDF 2016 guidelines on the management of infected wounds for patients with DM. Due to the lack of evidence, the therapeutic status of treatment options like hydrocolloid gels, NPWT, hyperbaric oxygen and aligate dressings could not be ascertained. There is a need to generate stronger evidence regarding the commonly used methods in the treatment of diabetic wound infections. The authors may refer to this latest literature to enhance their review; Aljerf *et al.* (2019 ^1^).

 The manuscript has however some grammatical mistakes which need to be improved. As a whole, the topic is interesting. The manuscript is well organized and the authors systematically addressed the issues. I recommend indexing of this article after minor revisions.

Is the work clearly and accurately presented and does it cite the current literature?

Yes

If applicable, is the statistical analysis and its interpretation appropriate?

Yes

Are all the source data underlying the results available to ensure full reproducibility?

Yes

Is the study design appropriate and is the work technically sound?

Yes

Are the conclusions drawn adequately supported by the results?

Yes

Are sufficient details of methods and analysis provided to allow replication by others?

Yes

Reviewer Expertise:

NA

I confirm that I have read this submission and believe that I have an appropriate level of expertise to confirm that it is of an acceptable scientific standard.

## References

[1] : Guidelines for the preparation and isolation of Radionuclides produced with In-house Cyclotrons Bombardments. *Open Journal of Chemistry*.2019;5(1) : 10.17352/ojc.000013 020-029 10.17352/ojc.000013

# Peer review: Reviewer response for version 1

**DOI:** 10.5256/f1000research.20803.r63395

**Published:** 2020-05-22

**Authors:** Nousheen Bibi

**Affiliations:** 1 Department of Bioinformatics, Shaheed Benazir Bhutto Women University, Peshawar, Pakistan

In the research article "Management of infected diabetic wound: a scoping review of guidelines" Huidi *et al. *have conducted a study and concluded that all guidelines and reviews have consistent suggestions on the assessment of the severity of infection, diagnosis, start, selection, and duration of antibiotic therapy. Overall the research work is well organized and presented. The methodology is sound and can be replicable. The author has concluded due to the lack of evidence, the therapeutic status of treatment options like hydrocolloid gels, NPWT, hyperbaric oxygen, and alligate dressings could not be ascertained. There is a need to generate stronger evidence regarding the commonly used methods in the treatment of diabetic wound infections.

Is the work clearly and accurately presented and does it cite the current literature?

Yes

If applicable, is the statistical analysis and its interpretation appropriate?

Not applicable

Are all the source data underlying the results available to ensure full reproducibility?

Yes

Is the study design appropriate and is the work technically sound?

Yes

Are the conclusions drawn adequately supported by the results?

Yes

Are sufficient details of methods and analysis provided to allow replication by others?

Yes

Reviewer Expertise:

My area of research is infectious diseases, neurology, and cancer biology. As a bioinformatician, I have expertise in data collection and analysis.

I confirm that I have read this submission and believe that I have an appropriate level of expertise to confirm that it is of an acceptable scientific standard.
